# Echocardiography-derived total atrial conduction time (PA-TDI duration): risk stratification and guidance in atrial fibrillation management

**DOI:** 10.1007/s00392-021-01917-9

**Published:** 2021-08-28

**Authors:** Patrick Müller, Bob Weijs, Nadine M. A. A. Bemelmans, Andreas Mügge, Lars Eckardt, Harry J. G. M. Crijns, Jeroen J. Bax, Dominik Linz, Dennis W. den Uijl

**Affiliations:** 1 Department of Cardiology II–Electrophysiology, University Hospital of Münster, 48149 Münster, Germany; 2 Department of Cardiology and Cardiovascular Research Institute Maastricht (CARIM), Maastricht University Medical Center+, Maastricht, The Netherlands; 3 Department of Cardiology and Electrophysiology, Katholische Stiftung Marienhospital Aachen, Aachen, Germany; 4 Cardiovascular Center, St. Josef Hospital Bochum, Ruhr-University Bochum, Bochum, Germany; 5 Cardiology and Angiology, Bergmannsheil, Ruhr University Bochum, Bochum, Germany; 6 Department of Cardiology, Leiden University Medical Center, Leiden, The Netherlands; 7 Department of Cardiology, Radboud University Medical Center, Nijmegen, The Netherlands; 8 Department of Biomedical Sciences, Faculty of Health and Medical Sciences, University of Copenhagen, Copenhagen, Denmark

**Keywords:** PA-TDI duration, Total atrial conduction time, Atrial fibrillation

## Abstract

Atrial fibrillation (AF) is a major cause of cardiovascular morbidity and mortality. To early detect and to avoid AF-related complications, several cardiac imaging modalities and approaches aim to quantify the severity of the underlying atrial cardiomyopathy (i.e., the extent of atrial remodeling). However, most established cardiac imaging modalities just incorporate single components of atrial remodeling and do not reflect the complete multifactorial process, which may contribute to their limited predictive value. Echocardiography-derived PA-TDI duration is a sophisticated echocardiographic parameter to assess total atrial conduction time and directly reflects both electrical and structural changes to the atria. Therefore, PA-TDI duration provides a more comprehensive quantification of the extent of atrial remodeling than other imaging modalities. In this article we review the role of PA-TDI duration as a marker of atrial remodeling and summarize the available data on PA-TDI duration to identify patients at risk for AF, as well as to guide AF management. Moreover, we discuss how to assess PA-TDI duration and provide recommendations on the implementation of PA-TDI duration into routine clinical care.

**Graphic abstract:**

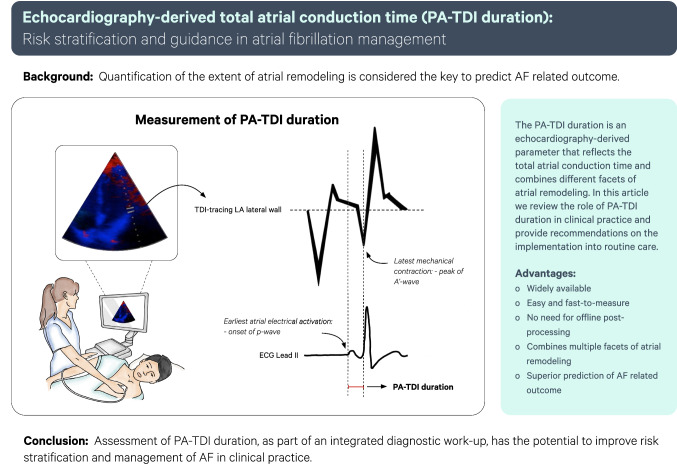

## Introduction

**Fig. 1:**
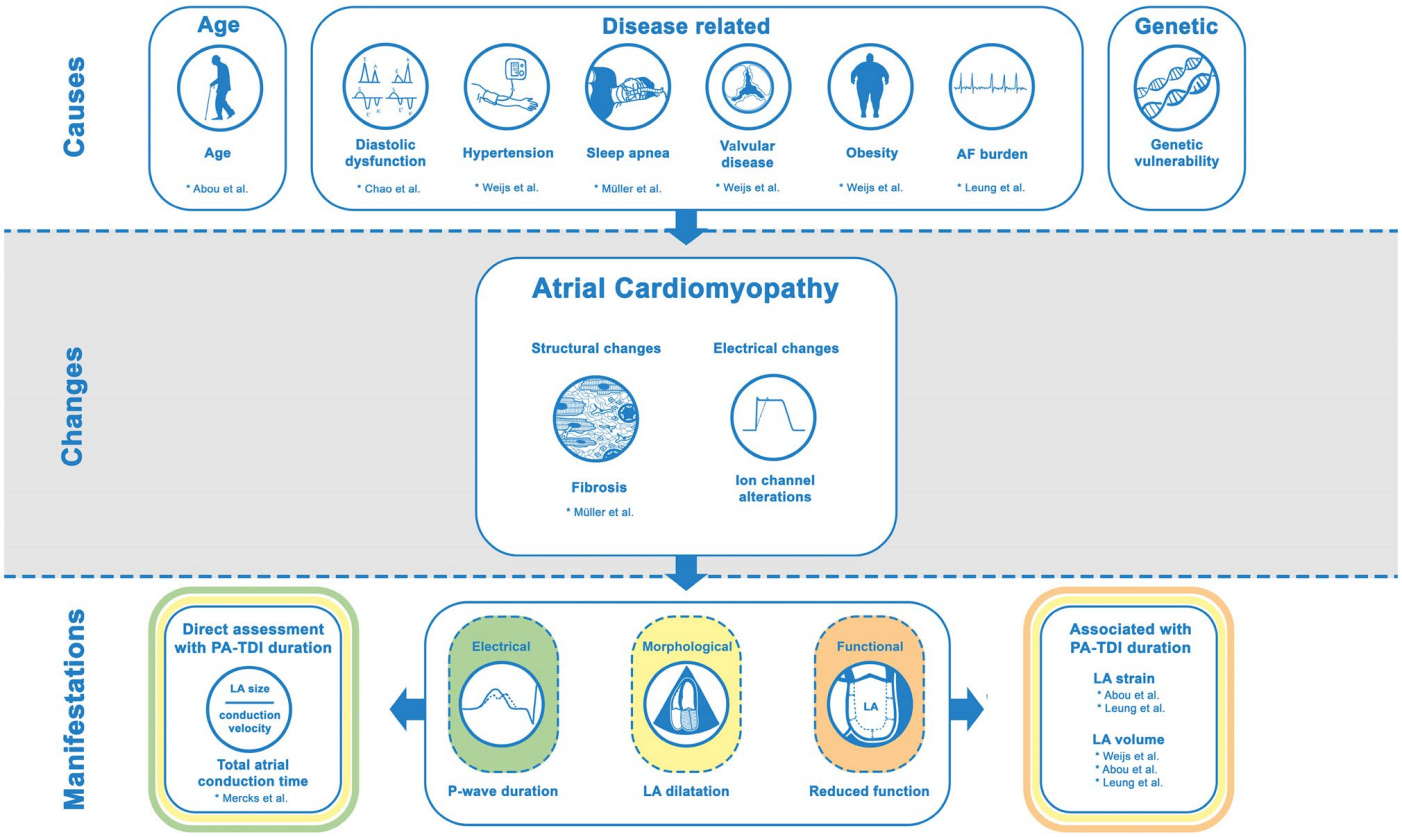
Role of PA-TDI duration in the visualization of atrial cardiomyopathy. The PA-TDI duration is associated with several conditions that can cause atrial cardiomyopathy. Moreover, the PA-TDI duration reflects the extent of atrial fibrosis, one of the processes involved in atrial cardiomyopathy. Finally, the PA-TDI duration allows direct assessment of both electrical and anatomical manifestations of atrial cardiomyopathy by estimating the total atrial conduction time and is strongly associated with the functional manifestations such left atrial (LA) dysfunction. *AF* atrial fibrillation

Atrial fibrillation (AF) is the most common sustained cardiac arrhythmia in clinical practice and affects more than 6 million people in Europe [1]. AF is often the result of an underlying atrial cardiomyopathy that can be caused by a variety of diseases and concomitant risk factors (e.g., hypertension, obstructive sleep apnea and obesity) that lead to structural (e.g., fibrosis) and electrical changes (e.g., ion channel alterations) to the atria: atrial remodeling [2, 3]. Subsequent clinical manifestations of atrial remodeling can be morphological, electrical and/or functional (Fig. 1) [4, 5].

Quantification of the extent of atrial remodeling is considered the key to predict the development and progression of AF, the response to AF treatment and the risk for AF related complications. However, most imaging modalities that are used to quantify atrial remodeling fail to capture more than one clinical manifestation of this process, thereby limiting their predictive value.

Echocardiography derived total atrial conduction time is a marker of atrial remodeling that reflects both morphological (atrial size) and electrical (conduction velocity) manifestations [6]. Total atrial conduction time is measured during sinus rhythm as the time interval from the beginning of the P-wave on the surface electrocardiogram (ECG) to the peak A’-wave on the Tissue-Doppler Imaging (TDI) tracing of the lateral left atrial (LA) wall on echocardiography: PA-TDI duration. The PA-TDI duration reflects the time needed for the atrial depolarization to conduct from the sinus node through the atrial tissue to the LA lateral wall and to result in an active contraction as measured with TDI. Measurement of the PA-TDI duration provides a more comprehensive estimation of the extent of atrial remodeling than other parameters [7].

Here, we review the role of the PA-TDI duration as a marker of atrial remodeling and summarize the available data on PA-TDI duration to detect patients at risk for AF, to guide AF treatment and monitor the effect of risk factor modification on the left atrial substrate. Importantly, we discuss how to assess PA-TDI duration and provide recommendations on the implementation of PA-TDI duration into routine clinical care.

## Echocardiographic assessment

**Fig. 2:**
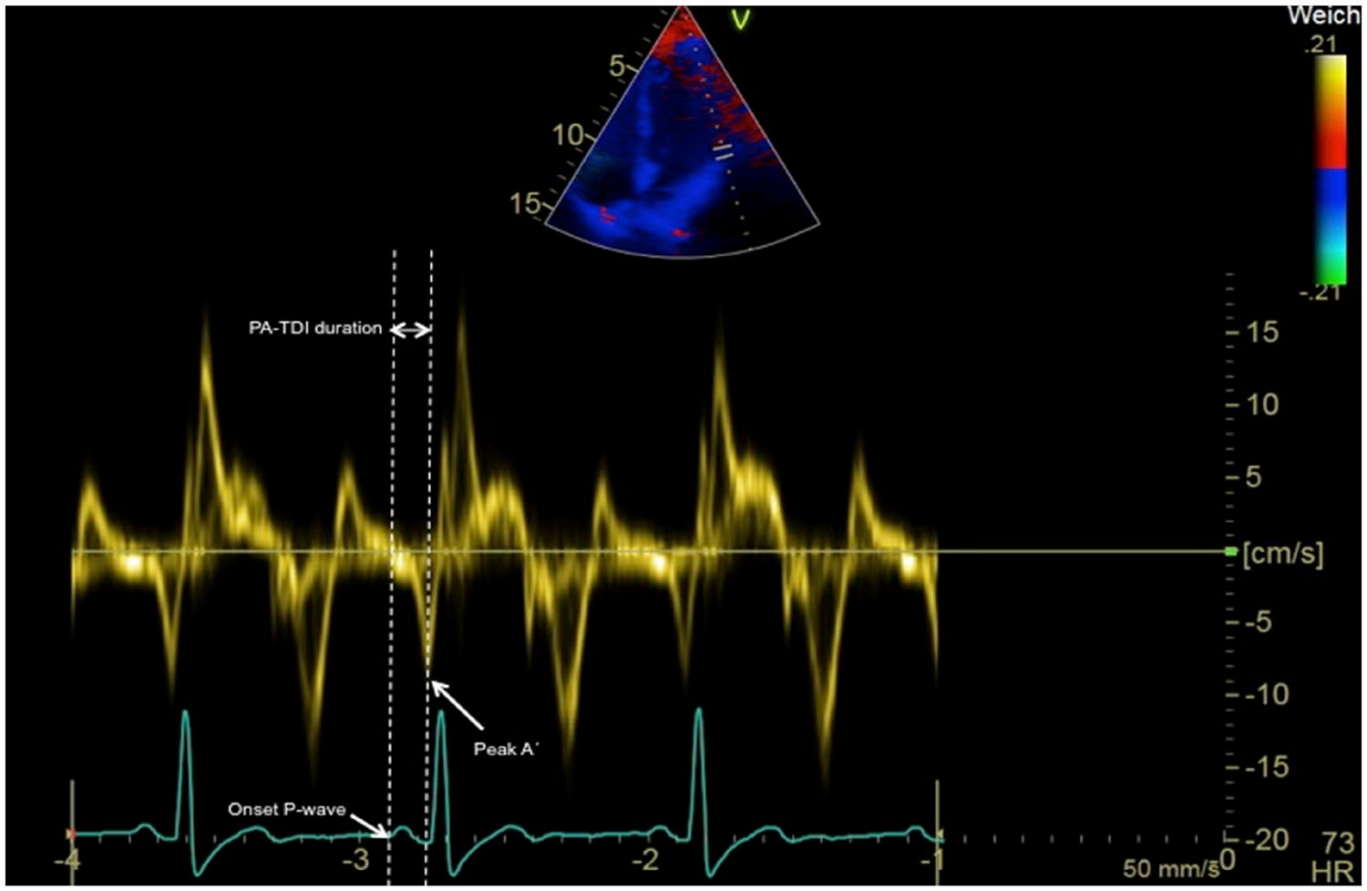
Measurement of the PA-TDI duration. An example of the measurement of total atrial conduction time via PA-TDI duration with 131.3 ms: as time interval from the onset of P-wave in lead II of the ECG to the peak A′-wave of the left lateral atrial wall

The PA-TDI duration is measured from ECG-gated pulse-wave TDI recordings of the apical four-chamber view using two-dimensional transthoracic echocardiography. Settings are optimized for the highest framerate as possible (at least > 115 frames/s, corresponding to a temporal resolution of 8.7 ms or smaller). A region of interest marker is placed at the LA lateral wall just above the mitral annulus providing the tracing of the mechanical activation in that area (Fig. 2)*.* The PA-TDI duration is measured during sinus rhythm as the time interval from the onset of the P-wave in lead II of the surface ECG (start of electrical depolarization) to the peak A’-wave on the tissue Doppler tracing of the LA lateral wall (active atrial contraction).

## Validation and determinants of PA-TDI duration

The use of PA-TDI duration to estimate total atrial conduction time has been validated by Merckx et al. using P-wave duration (PWD) on the signal-averaged ECG as the ‘gold standard’ (*R* = 0.911, *p* < 0.001) [6]. PWD in this study was measured as the time-interval between the onset and the offset of the P-wave (defined as the junction between the isoelectric line and the beginning of the P-wave deflection and the junction between the end of the P-wave deflection and the isoelectric line, respectively). Importantly, the authors found that assessment of PA-TDI duration was less time-consuming compared to measurement of PWD using signal-averaged ECG (1.0 ± 0.5 min vs 20 ± 5.0 min, respectively, *p* < 0.01).

Histological validation of PA-TDI duration to assess atrial remodeling was provided by Müller et al. who demonstrated the correlation between PA-TDI duration and the degree of atrial fibrosis inside the right atrial appendage, one of the hallmark processes involved in atrial remodeling [8]. In addition, various studies have demonstrated the prognostic impact of PA-TDI duration on the occurrence of new-onset AF, postoperative AF and AF recurrence after cardioversion or ablation, thereby implicitly strengthening its validity [7–25].

The PA-TDI duration is strongly influenced by factors that play a role in atrial remodeling (Fig. 1). In a group of 386 patients without structural heart disease, Abou et al. demonstrated that PA-TDI duration increases across all age categories, reflecting an age-related component of atrial remodeling [26]. These results are in agreement with work from Weijs et al. and Leung et al. who found a similar age-related impact on PA-TDI duration [27, 28]. Furthermore, Weijs et al. demonstrated that PA-TDI duration increases in the presence of hypertension, a history of AF, a higher body mass index and valvular disease [27]. Similarly, Chao et al. demonstrated that the PA-TDI duration significantly increases across the different stages of diastolic dysfunction [29]. The impact of obstructive sleep apnea on PA-TDI duration has been demonstrated by Müller et al. [30]. In this study the PA-TDI duration was higher in patients with obstructive sleep apnea compared to controls (131.4 ± 16.0 ms vs 120.0 ± 6.4 ms, *p* < 0.001). Furthermore, after effective treatment with continuous positive airway pressure therapy, which is one of the mainstay treatment options for obstructive sleep apnea patients [31], the PA-TDI duration decreased with a mean delta of 6.4 ± 5.7 ms [30]. This observation demonstrates the dynamic character of atrial remodeling as well as the potential positive impact of preventive measures. Moreover, it underlines the value of PA-TDI duration to capture these changes.

The PA-TDI duration shows a close correlation to conventional echocardiographic indices of atrial remodeling. Weijs et al. demonstrated that PA-TDI duration is associated with left atrial dilatation (i.e., larger LA diameter is associated with a longer PA-TDI duration) [27]. However, the same authors also demonstrated that in a group of patients with idiopathic AF a similar LA volume index was found compared to a group of healthy controls, whereas PA-TDI duration was prolonged, reflecting a preclinical underlying disease [32]. In addition, Abou et al. found that PA-TDI duration was inversely related to LA reservoir strain, reflecting a reduced LA compliance [26]. These observations were confirmed in a large study by Leung et al. comprising of 602 patients with AF and 342 controls, that demonstrated that a longer PA-TDI duration was associated with a larger LA volume index (higher extent of left atrial dilatation) and a reduced LA reservoir strain (reduced LA compliance or increased stiffness) [28].

## Clinical applications

### Prediction of new-onset AF

AF is associated with a higher cardiovascular morbidity and mortality, and timely identification of AF may help to avoid complications and improve outcome [33–35]. There is a considerable amount of data demonstrating the value of PA-TDI duration to predict the development of new-onset AF (Table 1A). Different studies have demonstrated that PA-TDI duration consistently and accurately predicts new-onset AF (1) in a general cardiology population; (2) in patients with heart failure; (3) after myocardial infarction; (4) in congenital heart disease; (5) in end-stage renal disease; (6) in patients with cryptogenic stroke; and (7) in patients with hypertrophic cardiomyopathy [11–13, 20, 21, 24, 25].

**Table 1:**
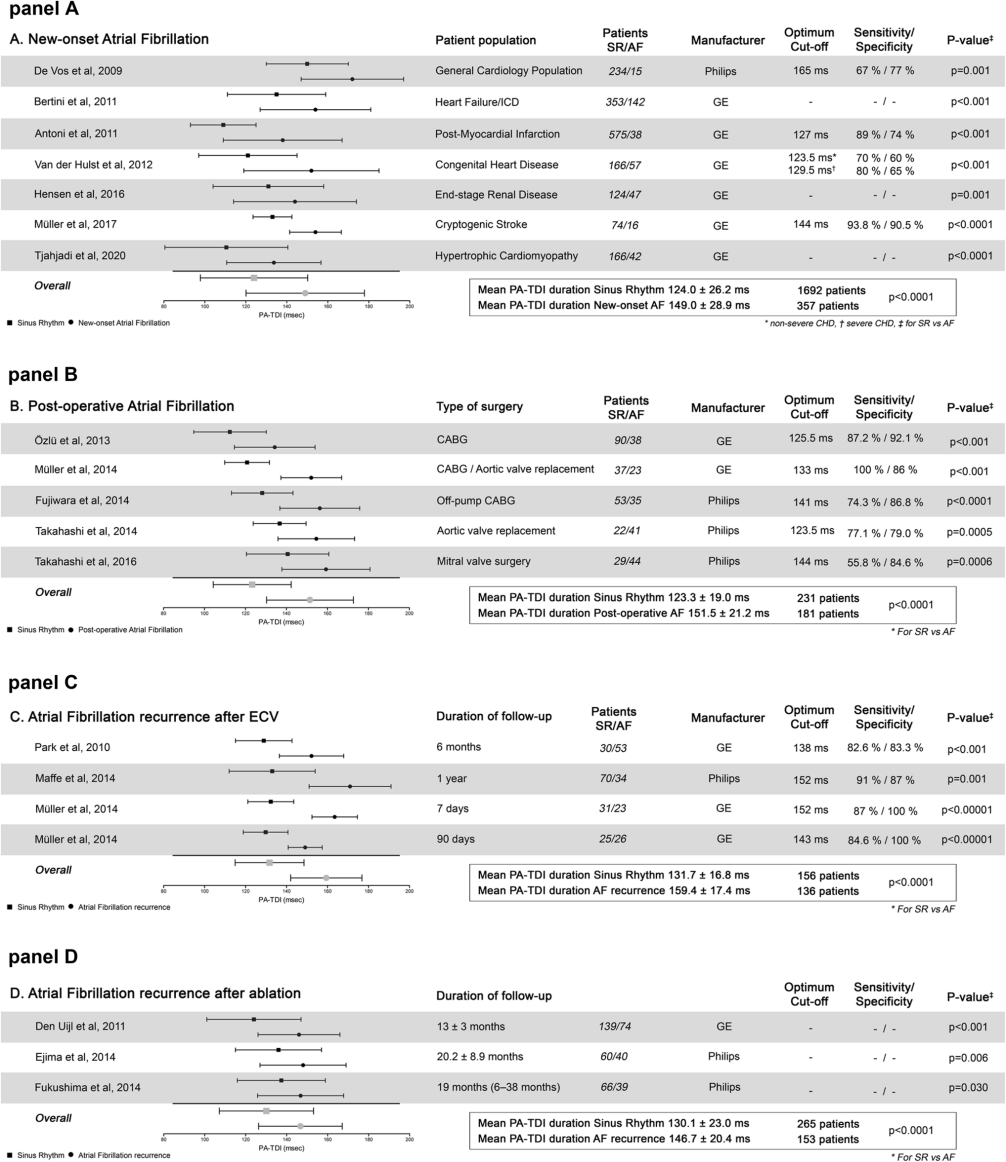
PA-TDI duration in different clinical scenarios

### Post-operative AF

Post-operative AF (POAF) is the most common sustained arrhythmia after cardiac surgery and is associated with an increased morbidity and mortality as well as prolonged in-hospital stay [36]. Pre-operative identification of patients at a high risk of POAF may help to guide pharmacological prophylaxis and reduce complications. Five studies have consistently shown that PA-TDI duration can be used to identify patients at high risk of POAF (Table 1B) [8, 18, 19, 22, 23]. Of note, PA-TDI duration was longer in patients after aortic valve replacement or mitral-valve surgery than in patients after bypass surgery only possibly reflecting valvulopathy related atrial remodeling.

### AF recurrence after rhythm control interventions

Antiarrhythmic drugs, electrical cardioversion and catheter ablation are the cornerstones of rhythm control therapy of AF. Despite significant advancement of these therapies, AF recurrence rates are high [37]. Age, co-morbidities and AF duration before restoration of sinus rhythm predispose an atrial remodeling process and leads to both the progression of AF and recurrence of AF after interventions for rhythm control strategies [4, 5, 38]. Overall, eight studies have looked at PA-TDI duration as a predictor of AF recurrence (Table 1C, D) [7, 9, 10, 14–17]. To summarize, four studies with 262 patients and four studies with 550 patients analyzed the predictive value of PA-TDI duration for AF recurrence after successful electrical cardioversion and catheter ablation, respectively. Of note, all these studies demonstrated the ability of PA-TDI duration to predict AF recurrence.

### Assessment of thromboembolic risk

AF predisposes patients to a fivefold increased risk of ischemic stroke [35]. Thus, individual thromboembolic risk stratification is desirable to identify candidates which most benefit of oral anticoagulation therapy. Recently, Leung and colleagues investigated the association between PA-TDI duration and ischemic stroke in 1361 patients with first diagnosis of AF [39]. Assessment of LA reservoir strain and PA-TDI duration on echocardiography after initial CHA2DS2-VASc scoring provides improved risk stratification for stroke over a mean of 7.9 years, especially for the low-to-intermediate risk groups. Importantly, PA-TDI duration was the strongest echocardiographic predictor of stroke in this study.

## Comparison to conventional echocardiography

Echocardiography is commonly used to quantify atrial remodeling by measurement of LA size and function. LA size and function have proven to be an important predictor of AF related outcome [38–40]. However, measurement of LA size using standard two-dimensional echocardiography is less accurate than with three-dimensional imaging modalities, such as MRI or CT: The shape of the LA is often asymmetrical, making it more difficult for two-dimensional imaging echocardiography to accurately assess true LA volume [41]. This limits the clinical applicability of echocardiography derived volumetric measurement, such as LA size and function, to predict AF related outcome. This is illustrated by the fact that PA-TDI duration has consistently demonstrated to be a stronger predictor of AF related outcome than LA size and function [7–12, 14–23].

A more sophisticated echocardiographic parameter that does not rely on volumetric measurements to quantify atrial remodeling is LA strain. LA strain uses Speckle tracking tissue deformation analysis to assess global LA function and has proven to be an independent predictor of AF related outcome [42]. However, LA strain analyses require time-consuming offline post-processing by manual adjustment of tracing of the LA borders [43]. This limits the routine use of LA strain in clinical practice and its reproducibility. Importantly, Leung et al. demonstrated that although LA reservoir strain was a predictor of stroke in patients with AF, PA-TDI duration was a stronger predictor than LA strain [39].

Measurement of PA-TDI duration is angle-independent and the TDI-tracings have a high temporal resolution (i.e., a high framerate compared to 2D echocardiography and strain analyses). Moreover, the PA-TDI duration can be measured directly on the echo machine and does not require time-consuming offline post-processing and has a low intra- and interobserver variability.

## Clinical implementation

The PA-TDI duration can be assessed on each echocardiographic machine with the ability to record TDI with ECG-gating. Therefore, this technique is widely available and easy to introduce into clinical practice. However, there are some challenges that need to be overcome. Currently, no normal values for PA-TDI duration have been established. There seems to be a large variation in values for PA-TDI duration across the different studies (Table 1A–D). Although no head-to-head comparisons were made, there is a significant difference in mean PA-TDI duration in studies using a Philips system compared to a GE system (145.1 ± 21.7 ms vs 126.4 ± 26.3 ms, respectively, *p* < 0.0001). Most likely, this could be explained by a different time delay between the ECG-signal and the echo-signal across the two systems [44]. In addition, the variation in the values for PA-TDI duration can be partly attributed to the different patient populations that were studied. Importantly, despite these variations, studies consistently demonstrated a very low intra-observer variability (range 1.6–1.8 ms) and inter-observer variability (range 1.7–2.6 ms) in PA-TDI duration measurement, underlining the high degree of reproducibility [7, 11, 12, 28]. Altogether, it seems reasonable that for clinical implementation of PA-TDI duration, each echocardiography laboratory must establish its own normal values and cutoff values, specific for their population and ultrasound system. In addition, P-wave duration on signal-averaged ECG can be used to calibrate and validate laboratory specific measurements.

## Discussion

Quantification of atrial remodeling is considered the key to predict the development of AF, the response to rhythm control therapies and the occurrence of AF related complications. The PA-TDI duration is an echocardiography-derived parameter that can be used to quantify the severity of atrial remodeling [6]. The advantage of PA-TDI duration over other parameters is that it combines electrical as well as morphological manifestations of atrial remodeling and, therefore, provides a more comprehensive assessment of atrial remodeling. However, PA-TDI duration does not capture all manifestations of atrial remodeling. For example, PA-TDI duration holds no information about atrial refractoriness, action potential duration or heterogeneity of conduction [2].

PA-TDI duration is very closely related to PWD, as both parameters are indices of the total atrial conduction time. However, whereas PWD reflects the time interval between the earliest and latest atrial electrical activation, PA-TDI duration reflects the time interval between earliest atrial electrical activation and the latest atrial mechanical contraction. Theoretically, PA-TDI duration, therefore, provides a more comprehensive assessment of atrial remodeling than PWD. Practically, measurement of PA-TDI duration is less time-consuming than PWD derived from signal-averaged ECG [6], and has a superior prognostic value over PWD derived from a 12-lead ECG [18–21].

Heart rate can have an important impact on cardiac physiology, for example on the QT-time. The impact of heart rate on PA-TDI duration has not been investigated extensively. Chao et al. found a weak correlation of − 0.270 between PA-TDI duration and heart rate, meaning that roughly 7% of variation in PA-TDI duration can be related to the heart rate [29]. However, recently, the impact of heart rate on PWD has been studied thoroughly: Toman et al. demonstrated that within a normal range of 60–75 bpm, the impact of heart rate on PWD was neglectable [45]. Nevertheless, future studies should be directed to investigate the association between of heart rate and PA-TDI duration.

## Future directions

Assessment of PA-TDI duration as a routine echo-lab parameter may substantially improve risk stratification and management of AF. The simple, quick and inexpensive measurement of PA-TDI duration enables its integration into everyday clinical practice. As PA-TDI duration reflects different aspects and dynamics of atrial cardiomyopathy, this parameter may be useful: (1) to identify patients at risk for AF; (2) to guide and monitor individualized rhythm-control therapy; and (3) to identify which patients with low CHA_2_DS_2_-VASc score may benefit from anticoagulation therapy. Carefully designed clinical trials are needed to evaluate these potential applications of PA-TDI duration.

## Conclusions

The PA-TDI duration is an easy and fast-to-measure echocardiographic parameter that combines information about parts of the structural, electrical and functional changes to the atria to reflect the different and dynamic facets of atrial remodeling. Assessment of PA-TDI duration, as part of an integrated diagnostic work-up, has a potential role to improve risk stratification and management of AF in clinical practice.
